# Knowledge, attitudes and practices related to hypertension among residents of a disadvantaged rural community in southern Zimbabwe

**DOI:** 10.1371/journal.pone.0215500

**Published:** 2019-06-25

**Authors:** Pugie Tawanda Chimberengwa, Mergan Naidoo, on behalf of the cooperative inquiry group

**Affiliations:** Discipline of Public Health Medicine, School of Nursing and Public Health, University of KwaZulu-Natal, Durban, South Africa; Agha Khan University, UNITED REPUBLIC OF TANZANIA

## Abstract

**Background:**

Hypertension contributes significantly to cardiovascular and renal diseases. It can be controlled by lifestyle modifications, however in poor communities there is lack of awareness, and treatment and control of hypertension is often poor. The aim of this study was to determine hypertension knowledge, attitudes and practices in a disadvantaged rural community in Matebeleland South province of Zimbabwe.

**Methods:**

We conducted a descriptive cross-sectional survey on hypertensive patients in the community. A pre-tested and validated interviewer-administered questionnaire was used to collect demographic, awareness, treatment and control data among consenting hypertensive patients.

**Results:**

304 respondents were enrolled into the study (mean age, 59 years), and a majority were women (65.4%). The treatment default rate was 30.9%, and 25% of respondents on medication did not know their blood pressure control status. Knowledge on hypertension was poor, with 64.8% of respondents stating that stress was its main cause, 85.9% stated that palpitations were a symptom of hypertension and 59.8% of respondents added salt on the table. The more education respondents had received, the more likely they were knowledgeable about hypertension (odds ratio for secondary education, 3.68 [95% CI: 1.61–8.41], and for tertiary education, 7.52 [95% CI: 2.76–20.46], compared to those without formal education). Those who believed in herbal medicines (50.7%) and those who used traditional medicines (14.5%) were 53% (95% CI: 0.29–0.76) and 68% (95% CI: 0.29–0.76) less likely to be knowledgeable about hypertension compared to those who did not believe in or use traditional medicines, respectively.

**Conclusion:**

Members of the community had poor knowledge on hypertension. This was associated with a lack of education and with strong beliefs in herbal and traditional medicines in the community, which influenced attitudes and practices on hypertension. Dietary risk factors were linked to poor knowledge. Hypertensive medicine shortages at the clinic resulted in worsened hypertension care and poor hypertension outcomes in the community.

## Introduction

Hypertension, the most common incidentally diagnosed chronic disease, is a major risk factor for cerebro-vascular accidents as well as coronary heart diseases, with two-thirds of all cerebro-vascular accidents attributable to poor hypertension control [1,2]. Together with other cardiovascular diseases, these public health problems that are strongly linked to urbanization, aging populations, westernized socio-economic sedentary lifestyles promoting excessive salt and alcohol intake, smoking, obesity as well as lack of physical exercise [3–6].

While hypertension is prevalent in both high income and low- and middle-income countries (LMICs), 80% of deaths due to cardiovascular diseases occur in LMICs [4,7]. Hypertension is a particularly significant health challenge in LMICs that are experiencing epidemiological transition from communicable to non-communicable diseases [8–10], such as Zimbabwe. There is a shortage of national data on hypertension prevalence studies in Zimbabwe, but the Zimbabwe STEPwise survey demonstrated that in 2005, the national hypertension prevalence was 27% (23,2% among males and 29% among females) [11]. In this study, an average prevalence of 17.9% was recorded among three other provinces (outside our survey province) focusing on both urban and rural settings [11]. A study that summarized hypertension prevalence over a 14-year period from 1997 to 2010 estimated the pooled prevalence of hypertension in Zimbabwe at 30% [12]. In a hypertension study done in Bulawayo city, in southern Zimbabwe, the highest prevalence of 38.4% was reported [13].

Several factors affect prevention, diagnosis, treatment and control of hypertension [14]. The most important barrier to diagnosis is lack of knowledge and awareness on hypertension and its complications [15]. Almost half of hypertension-related deaths can be averted with compliance or adherence to antihypertensive treatment [7]. Patient education is a key component in the programs and interventions designed to control hypertension, so it is therefore important to assess the patients’ knowledge and awareness of hypertension [16]. Efforts to control hypertension have included improving public knowledge and awareness on the risks and complications of hypertension [16]. The aim of this study was to determine the level of knowledge, attitudes and practices with regards to hypertension in ward 14, Gwanda district (Zimbabwe) as part of a community based participatory research (CBPR) we carried out. This paper will report on the quantitative baseline findings on the community’s knowledge and awareness on hypertension.

## Methods

### Study design

We conducted a baseline descriptive cross-sectional study to evaluate knowledge, awareness and perceived control of hypertension among patients living with hypertension in the community.

### Study setting

The survey was undertaken in Ward 14, a rural area located about 50 km south-west of Gwanda town, Matebeleland South province in Zimbabwe. The Gwanda district population at the time of the survey was 115 778 inhabitants, which was 16.9% of the provincial population, and Ward 14 had 1384 households, 5867 inhabitants of which 55% were females [17].

### Participants

All persons above the age of 18 years who reported having been diagnosed with hypertension, regardless of whether they were taking anti-hypertensive medication or not, and resident in Ward 14 qualified to be enrolled into the study. They included patients seen at Sengezane rural health center and individuals identified in the villages, schools and business premises, as well as hypertensive patients who were seen at Gwanda provincial hospital (primary referral center for Sengezane clinic) whose address originated from Ward 14.

The exclusion criteria were any person who was hypertensive not consenting to the study and those below the age of 18 years.

### Sampling and sample size calculations

We used the Dobson formula to determine sample size n = (z^2^pq)/Δ^2^, where n = sample size, z = standard error risk, p = prevalence of hypertension (among people living with hypertension), q = 1-p (proportion of people without hypertension) and Δ = absolute precision. Assuming 95% CI (z = 1.96), a prevalence of hypertension (p) of 27% [11], and using a precision of 5%, it was established that an adequate sample size would comprise 303 people living with hypertension.

### Ethics approval and consent to participate

All phases of this research were jointly approved by the Medical Research Council of Zimbabwe (MRCZ/A/2136) and the Biomedical Research Council of the University of Kwa-Zulu Natal, South Africa (BFC318/16). Authority was sought from the Ministry of Health and Child Care Zimbabwe through the Provincial Medical Director, Matebeleland South and the District Medical Officer for Gwanda. Gatekeeper’s authority was sought from the Ministry of Local Government at the offices of District Administrator. The Chiefs, headmen, religious leaders, health center committee and community advisory board were approached through a Ward 14 councilor. Written informed consent was obtained from all research participants.

### Data collection

Quantitative data was collected using an interviewer-administered questionnaire (S1 File) which was translated into isiNdebele language (S2 File) for respondents who could not speak/ understand English language.

Data collection was done by cooperative inquiry group (CIG) members. These comprised of hypertensive patients, community leaders and village health workers. They were joined by two nurses and the principal investigator. The CIG members were trained on quantitative data collection using interviewer administered questionnaires. The focus was on hypertension knowledge, awareness, treatment and control, recording of data for standardization and uniformity including ethical issues. The principal investigator developed the interviewer administered questionnaire which was discussed, validated and adopted by the CIG for data collection. The validation of the tool was done during CIG meetings where the participants discussed and understood the meaning of each of the questions in the data collection tool, recording and interpretation of the responses. Each CIG member was given five copies of the questionnaire to use as a pre-test and responses were shared and agreed upon. Thereafter, a two-week-long data collection exercise was conducted from May 30 to June 9, 2017. Data collected included respondents’ demography, risk factors for hypertension, knowledge, attitudes, perceptions and barriers to treatment compliance.

### Statistical methods

Frequencies and proportions were calculated for respondents’ demographic profiles, lifestyle related factors, beliefs and knowledge on hypertension treatment and control. To conduct a regression analysis, questions were re-coded, scored, aggregated into points and clustered on knowledge, attitude and practice. The survey had ten knowledge questions on hypertension and scoring six or more points by a respondent was considered good knowledge while five or less points was poor knowledge. The survey had eight hypertension practice questions and scoring five points or more was good practice while less than five points was poor practice. The survey had five questions on participant attitudes on hypertension and scoring three or more points was deemed good attitude while less than 3 points was regarded as poor. A mean score was used to assess whether the patient had good knowledge or poor knowledge, attitude and practice likewise. After classifying the participants into the above categories, good knowledge as cases and poor knowledge we calculated the odds ratio to determine the association with independent variables (demographic, beliefs and lifestyle factors), the same was applied to attitude and practice. Factors with a p value < 0,1 on bivariate analysis were then entered into the logistic regression model. Data was analyzed using Microsoft Excel, Stata and Epi-Info 7 software.

### Study validity and bias

Selection bias was reduced by ensuring high participation rates and interviewer bias was reduced by using trained CIG members who had standardized questionnaires. In instances, where clinic records were available, they were utilized for triangulation and to reduce information bias.

## Results

A total of 304 people living with hypertension participated in the study and the mean age of the participants was 59 (Q1-Q3; 46–72) years. Table 1 shows the sociodemographic data for hypertensive patients who were enrolled into the study.

**Table 1:** Socio-demographic data and lifestyle related factors for people living with hypertension in ward 14, Gwanda district; community-based action research project, May 2017 Demographic profiles of respondents.

Characteristic	Frequency, n (%)
Gender	
*Male*	108 (35.5)
*Female*	196 (64.5)
Marital status	
*Single*	**30 (9.9)**
*Married*	**179 (58.9)**
*Widowed*	**71 (23.4)**
*Divorced*	**24 (7.9)**
Religion	
*Apostolic*	7 (2.3)
*African religion*	37 (12.2)
*Christianity*	257 (84.5)
*Muslim*	2 (0.7)
*Other*	1 (0.3)
Level of education (n = 303)	
*None*	33 (10.9)
*Primary*	122 (40.3)
*Secondary*	95 (31.4)
*Tertiary*	53 (17.5)
Job description (n = 144)	
*Skilled*	56 (38.9)
*Unskilled*	88 (61.1)
Monthly income (US$)	
*<100*	40 (13.2)
*100–300*	43 (14.1)
*>300*	63 (20.7)
*Not declared*	158 (52.0)
**Life style related factors of participants**	
**Factor**	**Frequency, n (%)**
Ever consumed alcohol	76 (25.0)
Alcohol consumption (n = 76)	
*1–3 days/month*	26 (34.2)
*1–4 days/week*	25 (32.9)
*5+ days/week*	12 (15.8)
*Less than once per month*	13 (17.1)
Ever smoked cigarettes	36 (11.8)
Current cigarette smoker (n = 36)	15 (41.7)
Fruit consumption for more than 3 days a week	224 (73.7)
Vegetables consumption for more than 3 days a week	296 (97.4)
Adding salt on table	179 (58.9)
Cooking oil source	
*Margarine*	1 (0.3)
*Peanut butter*	38 (12.5)
*Vegetable oil*	264 (86.8)

The study sample consisted of 64.5% females and the respondents were predominantly Christians (84.5%). Fifty one percent attended primary level or below with about 11% having not attended primary school. Seventy five percent of respondents did not drink alcohol and only 12% had ever smoked. Twenty four percent ate fruits on more three days per week, while 60% reportedly added salt to their food at the table.

### Knowledge and beliefs on hypertension

Table 2 shows the respondents’ knowledge and beliefs on HT treatment and control among respondents.

**Table 2:** Knowledge and beliefs on hypertension, treatment and control among hypertensive patients in Ward 14, Gwanda district community-based action research project on hypertension, May 2017.

Knowledge category	Frequency, n (%)
Positive family history of hypertension	204 (67.1)
Positive family history of known hypertension complications	116 (39.3)
Belief in effectiveness blood pressure pills to manage hypertension	285 (93.8)
Belief in herbal remedies to manage hypertension	153 (50.7)
Agree to using African medicines to manage hypertension	44 (14.5)
Can use traditional medicines to control your blood pressure	44 (14.5)
Source of knowledge on high blood pressure	
*Local clinic nurse*	172 (56.6)
*Village health worker*	35 (11.5)
*Public hospital*	55 (18.1)
*Private doctor*	33 (10.9)
*Other*	9 (3.0)
Has a health worker / village health worker ever discussed with you about blood pressure treatment and control?	133 (43.8)
What are the causes of blood pressure?	
*Drugs*	16 (5.3)
*Old age*	23 (7.6)
*Stress*	197 (64.8)
*Witchcraft*	18 (5.9)
*Unknown*	48 (145.8)
*Other*	2 (0.7)
What are the signs and symptoms of high blood pressure?	
*Asymptomatic*	22 (7.2)
*Headache*	1 (0.3)
*Palpitations*	261 (85.9)
*Other*	8 (2.6)
*Don’t know*	12 (4.0)
Can one have high blood pressure without any signs and symptoms?	161 (53.0)
What are the consequences of untreated blood pressure?	
*Stroke*, *Heart failure*, *Kidney failure*	278 (91.4)
*Other*	7 (2.3)
*Don’t know*	19 (6.3)
*What are the risk factors for developing high blood pressure*?	
*Hereditary*	10 (3.3)
*Diet*	253 (83.2)
*Don’t know*	41 (13.5)

Family history of hypertension was reported by 67% of respondents. Ninety four percent believed tablets lower blood pressure, 51% also believed in the use of traditional remedies and 15% used medicine from traditional healers. The local clinic nurse was primarily the source of knowledge on hypertension for 57% of the respondents while the village health workers were reportedly consulted by only 12%. Stress was reported as the commonest cause (64.8%) of hypertension, palpitations were the commonest symptom of HT (85.9%) and diet was reported as the commonest risk factor for hypertension (83.2%).

### Attitudes and practices on hypertension

Table 3 shows the attitudes and practices of respondents on the control of hypertension, their preferred service providers and access areas for follow up.

**Table 3:** Attitudes and practices on treatment and control of hypertension in Ward 14, Gwanda district community-based action research project on hypertension, May 2017.

Treatment and control	Frequency, n (%)
How do you currently control your blood pressure?	
*Blood pressure tablets*	260 (85.5)
*Traditional medicines*	27 (8.8)
*Prayer*	9 (3.0)
*Other*	8 (2.6)
Those currently taking medication for hypertension	250 (82.2)
Taken medication regularly in the past 2 weeks (n = 250)	229 (91.6)
Those having challenges with taking hypertensive treatment	58 (19.1)
Preferred place of followed up for hypertension management	
*Local clinic*	159 (52.3)
*Public hospital*	48 (15.8)
*Private doctor*	49 (16.1)
*My home*	13 (4.3)
*Other*	3 (1.0)
*Not stated*	32 (10.5)
When last was your blood pressure checked?	
*< 1 month ago*	152 (50.0)
*2–4 months ago*	63 (20.7)
*> 4 months ago*	58 (19.1)
*Missing*	31 (10.2)
Is your blood pressure well controlled?	
*Yes*	196 (64.5)
*No*	32 (10.5)
*Don’t know*	46 (15.1)
*Not stated*	30 (9.9)
Ever defaulted hypertensive medication	94 (30.9)
Reasons for defaulting treatment (n = 93)	
*Had developed side effects from medicines*	11 (11.8)
*Tablets make me sick*	7 (7.5)
*To avoid addiction*	11 (11.8)
*Treatment was not effective*	14 (15.1)
*Trying alternative remedies*	16 (17.2)
*Feeling much better*	26 (28.0)
*Other*	8 (8.6)
Agreed to use of traditional medicines to control blood pressure?	44 (14.5)

The majority of the respondents (85.5%) reported that they were using BP tablets to control hypertension while 8.8% used African traditional medicines. The commonest places where respondents were being followed up for continued care were the local clinic (52.3%), the private doctor (16.1%) and the public hospital (15.8%). Sixty four percent of respondents stated that they had well-controlled blood pressure and 25% did not know whilst 31% reported to have had defaulted treatment. Feeling much better (27.6%) and assuming treatment was not effective (14.9%), were some excuses given for defaulting treatment.

### Regression analysis

Table 4 shows a logistic regression analysis of factors affecting knowledge on hypertension.

Data was re-coded such that those who had scored six or more points out of ten had good knowledge, scoring five or more out of eight had good practice, and scoring three or more out of five was deemed good attitude towards hypertension treatment and control.

**Table 4:** Regression analysis of factors affecting hypertension knowledge in Ward 14, Gwanda district community-based action research project on hypertension, May 2017.

Factor	Hypertension knowledge		OR (95% CI)
	good (n = 196)	poor (n = 108)	
Gender			
*Female*	125	71	Ref
*Male*	71	37	1.09 (0.67–1.78)
Religion			
*Apostolic*	6	1	3.23 (0.38–27.28)
*African religion*	20	17	0.63 (0.32–1.27)
*Christianity*	167	90	Ref
*Muslim/other*	3	0	-
Level of education (n = 303)			
*None*	13	20	Ref
*Primary*	71	51	2.14 (0.98–4.70)
*Secondary*	67	28	3.68 (1.61–8.41) **
*Tertiary*	44	9	7.52 (2.76–20.46) ***
Job description (n = 144)			
*Skilled*	45	11	Ref
*Unskilled*	59	29	0.50 (0.22–1.10)
Adding salt on table	110	69	0.72 (0.45–1.17)
Family history of hypertension	133	71	1.10 (0.69–1.81)
Family history of hypertension complications	78	38	1.11 (0.68–1.83)
Belief in blood pressure pills	187	98	2.12 (0.83–5.39)
Belief in herbal remedies	86	67	0.47 (0.29–0.76) **
Agree to using African medicines	18	26	0.32 (0.17–0.61) **
Source of knowledge on high blood pressure			
*Local clinic nurse*	107	65	0.51 (0.25–1.02)
*Village health worker*	18	17	0.33 (0.13–0.81) *
*Public hospital*	42	13	Ref
*Private doctor*	29	4	2.24 (0.66–7.57)
*Other*	0	9	-
How do you currently control your blood pressure?			
*Blood pressure tablets*	176	84	Ref
*Traditional medicines*	10	17	0.28 (0.12–0.64) *
*Prayer*	5	4	0.60 (0.16–2.28)
*Other*	5	3	0.80 (0.19–3.41)
Taken medication regularly in the past 2 weeks (n = 250)	152	78	0.89 (0.40–1.96)
Challenges with taking hypertensive treatment	36	22	0.79 (0.43–1.45)
Place preferred being followed up for hypertension management			
*Local clinic*	107	52	Ref
*Public hospital*	33	15	1.07 (0.53–2.14)
*Private doctor*	36	16	1.35 (0.66–2.75)
*My home*	5	8	0.30 (0.09–0.97) *
*Other*	1	2	0.24 (0.02–2.74)
*Not stated*	14	18	0.38 (0.17–0.82) *
Last blood pressure check			
*< 1 month ago*	109	43	Ref
*2–4 months ago*	37	26	0.56 (0.30–1.04)
*> 4 months ago*	37	21	0.70 (0.37–1.32)
*Missing*	13	18	0.28 (0.13–0.63) *
Blood pressure controlled			
*Yes*	135	61	Ref
*No*	24	8	1.36 (0.58–3.19)
*Don’t know*	25	21	0.54 (0.28–1.03)
*Not stated*	12	18	0.30 (0.14–0.66) **
Ever defaulted medication	58	36	0.72(0.43–1.22)
**Factor**	**Attitude**		**OR (95% CI)**
	good(n = 266)	poor(n = 38)	
Gender			
*Female*	175	21	Ref
*Male*	91	17	0.64 (0.32–1.28)
Religion			
*Apostolic*	6	1	0.53 (0.06–4.65)
*African religion*	21	16	0.12 (0.05–0.26) ***
*Christianity*	236	21	Ref
*Muslim/other*	3	0	-
Level of education (n = 303)			
*None*	24	9	Ref
*Primary*	108	14	2.89 (1.12–7.46) *
*Secondary*	84	11	2.86 (1.06–7.71) *
*Tertiary*	49	4	4.59 (1.28–16.44) *
Job description (n = 144)			
*Skilled*	51	5	Ref
*Unskilled*	75	13	0.57 (0.19–1.68)
Adding salt on table	150	29	0.40 (0.18–0.88) *
Family history of hypertension	176	28	0.70 (0.32–1.50)
Family history of hypertension complications	102	14	1.07 (0.53–2.18)
Belief in blood pressure pills	261	24	30.45 (10.10–91.79) ***
Source of knowledge on high blood pressure			
*Local clinic nurse*	159	13	1.17 (0.36–3.84)
*Village health worker*	30	5	2.39 (0.96–5.95)
*Public hospital*	46	9	Ref
*Private doctor*	30	3	1.96 (0.49–7.82)
*Other*	1	8	0.02 (0.00–0.22) **
Currently control your blood pressure			
*Blood pressure tablets*	244	16	Ref
*Traditional medicines*	9	18	0.03 (0.01–0.08) ***
*Prayer*	9	0	-
*Other*	4	4	0.07 (0.01–0.29) ***
Taken medication regularly in the past 2 weeks	216	14	7.41 (3.58–15.33)
Challenges with taking hypertensive treatment	47	11	0.18 (0.07–0.48) **
Last blood pressure check			
*< 1 month ago*	145	7	Ref
*2–4 months ago*	60	3	0.97 (0.24–3.86)
*> 4 months ago*	43	15	0.14 (0.05–0.36) ***
*Missing*	18	13	0.07 (0.02–0.19) ***
Blood pressure controlled			
*Yes*	189	21	Ref
*No*	29	9	0.36 (0.09–1.46)
*Don’t know*	31	6	0.08 (0.03–0.20) ***
*Not stated*	17	2	0.05 (0.02–0.14) ***
Ever defaulted medication	191	19	2.55 (1.28–5.08) **
**Factor**	**Practice**		**OR (95% CI)**
	good(n = 131)	poor(n = 173)	
Gender			
*Female*	71	125	Ref
*Male*	60	48	2.20 (1.36–3.55) **
Religion			
*Apostolic*	4	3	1.75 (0.38–8.00)
*African religion*	15	22	0.90 (0.44–1.81)
*Christianity*	111	146	Ref
*Muslim/other*	1	2	0.66 (0.06–7.35)
Level of education (n = 303)			
*None*	7	26	Ref
*Primary*	42	80	1.95 (0.78–4.87)
*Secondary*	49	46	3.96 (1.57–9.99) **
*Tertiary*	32	21	5.66 (2.08–15.38) **
Job description (n = 144)			
*Skilled*	37	19	Ref
*Unskilled*	37	51	0.37 (0.19–0.75) **
Family history of hypertension	99	105	2.00 (1.21–3.31) **
Family history of hypertension complications	58	58	1.59 (0.99–2.56)
Belief in blood pressure pills	128	157	4.35 (1.24–15.25)
Belief in herbal remedies	62	91	0.81 (0.51–1.28)
Agree to using African medicines	14	30	0.57 (0.29–1.13)
Source of knowledge on high blood pressure			
*Local clinic nurse*	66	106	0.60 (0.33–1.11)
*Village health worker*	15	20	0.72 (0.31–1.70)
*Public hospital*	28	27	Ref
*Private doctor*	22	11	1.93 (0.79–4.73)
*Other*	0	9	-
Currently control your blood pressure			
*Blood pressure tablets*	127	133	Ref
*Traditional medicines*	3	24	0.13 (0.04–0.45) **
*Prayer*	0	9	-
*Other*	1	7	0.15 (0.02–1.23)
Taken medication regularly in the past 2 weeks	122	108	8.16 (3.89–17.16) ***
Challenges with taking hypertension treatment (n = 252)	22	36	0.56 (0.31–1.03)
Place preferred being followed up for hypertension management			
*Local clinic*	69	90	Ref
*Public hospital*	22	26	1.10 (0.58–2.11)
*Private doctor*	35	14	3.26 (1.63–6.53) **
*My home*	2	11	0.24 (0.05–1.11)
*Other*	0	3	-
*Not stated*	3	29	0.13 (0.04–0.46) **
Last blood pressure check			
*< 1 month ago*	86	66	Ref
*2–4 months ago*	33	30	0.84 (0.47–1.52)
*> 4 months ago*	9	49	0.14 (0.06–0.31) ***
*Missing*	3	28	0.08 (0.02–0.28) ***
Blood pressure controlled			
*Yes*	110	86	Ref
*No*	12	20	0.47 (0.22–1.01)
*Don’t know*	6	40	0.12 (0.05–0.29) ***
*Not stated*	3	27	0.09 (0.03–0.30) ***
Ever defaulted medication (n = 272)	40	54	0.76 (0.46–1.25)

Key:

*Significant at α = 0.05

**Significant at α = 0.01

***Significant at α = 0.001

Ref = Reference against which other categories were measured against

Those who attained tertiary education and secondary education were 7.52 (95%CI:2.76–20.46) and 3.68 (95%CI;1.61–8.41) more likely to have better knowledge than those who had no formal education respectively. Those that believed in herbal medicines and those that used African traditional medicines were 53% (95% CI:0.29–0.76) and 68% (95% CI:0.29–0.76) less likely to have good knowledge compared to those who did not believe and use traditional medicines respectively. Attaining secondary education and tertiary education is 2.86 (95% CI: 1.06–7.71) and 4.59 (95% CI: 1.28–16.44) more likely to have a positive attitude towards hypertension as compared to respondents that have not received formal education. Those that added salt on the table were 60% (95% CI: 0.18–0.88) less likely to have their blood pressure controlled compared to those that did not. With regards to practice, those with a family history of hypertension were 2.00 (95% CI: 1.21–3.31) more likely to have their blood pressure controlled while those that took medication regularly in the preceding two weeks were 8.16 (95% CI: 3.89–17.16) more likely to have their blood pressure controlled.

## Discussion

This study was conducted in a disadvantaged rural area where formal education is low and more than half of the participants (51%) were not educated beyond primary school and 11% had no formal education at all. Educational attainment was directly proportional to knowledge on hypertension as those with tertiary education had better knowledge as compared to those without formal education. In a community where formal education is low and the persons afflicted by disease are vulnerable due to socio-economic factors, poor health seeking behaviors are common. Poverty, ignorance, a poor educational background and weak community health education platforms were determinants of poor knowledge.

There was generally poor knowledge on the risk factors, causes and awareness on hypertension among people living with hypertension. Most respondents (65%) believed that hypertension was caused by stress and only 17% knew that the cause is largely unknown. Diet (83%) was singled out as the commonest risk factor in developing hypertension, and 14% had no knowledge of risk factors for hypertension. Vegetable consumption was high on four or more days a week according to many of the respondents (67%) as they were the commonly available for consumption while other alternatives (such as meat) were scarce in the community. The consumption of fruits was low as they relied mainly on seasonal wild fruits. However, there was an identified risk of consuming too much salt with food (59%) in the community, and this was due to lack of knowledge on the risk factors of hypertension. The Zimbabwe’s National Health Strategy (2009–2013) reported the increase in hypertension prevalence is mainly attributed to high salt diet, lack of exercise, tobacco smoking and excess alcohol intake [18].

Hypertension rarely has attributable signs and symptoms in the early stages and many people go undiagnosed [2]. Only 7% of respondents correctly stated that it was asymptomatic while 86% incorrectly reported palpitations were the commonest symptom of hypertension. The lack of symptoms for patients with hypertension contributes to both lack of awareness and reduced compliance to treatment. The improvement in hypertension control cannot be measured by symptom relief, thus there is no perceptive benefit for the individual without knowledge. Knowledge on hypertension in the community should focus on primary prevention as this is cost effective in low-resource settings. It is known that hypertension awareness in Zimbabwe is low and this has an impact in low diagnosis, treatment and control hence there is need for a specific policy for prevention and control of hypertension in Zimbabwe [19,20]. Evidence has shown that even in high income countries, hypertension awareness remains a challenge, with 50% of the population being aware of their status and even fewer (an estimated to be 40%) aware of their status in LMIC [20–22].

Although significant progress has been made in improving hypertension awareness, treatment and control among patients living with hypertension, socio-economic disparities influence the level of hypertension awareness, treatment and control in LMIC [23]. In this community, there is little money to spend on health based on observations and declaration of income. Similarly, low financial incomes could also explain the reduced pattern for abuse of alcohol and tobacco in the community. Most of the respondents were poor, and out-of-pocket health financing was a huge challenge. Several studies indicate that most Africans pay out-of-pocket for their health bills, and their care is supplemented by free services subsidized by donors and local governments. However, most of these resources are channeled towards communicable diseases leaving NCDs with little funding [24]. Thus, those diagnosed may not have access to treatment and may not be able to successfully control their illness over the long term due to poverty.

Hypertension affects populations negatively in low- and middle-income countries where health systems are weak [1,2,25]. Primary prevention of hypertension reduces the expenses on medical care and the resultant complications of high blood pressure. Awareness screening programs, skills training and capacity building of health workforce on how to deal with hypertension and its associated risk factors, including access to low-cost antihypertensive medicines, are key for developing countries with limited resources [26]. It was noted that 65% of those who took medication perceived that they had well-controlled blood pressure however we found out that their scale of measurement was based on experiencing or perceived “complications” rather than blood pressure readings.

Value beliefs and practices of an ethnic or racial group within a community can influence acceptance and adherence to health messages as advised by clinicians and academic researchers [27]. Although the majority (94%) believed in using tablets for controlling hypertension, there were strong traditional beliefs that the use of herbs (51%) and traditional medicines (15%) influenced the community’s health seeking behavior. We therefore proved that those who had belief in herbs and used traditional medicines had poor knowledge on hypertension. This has a potential bearing on the perpetuation of myths and misconceptions on hypertension treatment and control. This information provides an opportunity for correcting existing myths, misconceptions and misinformation for improved hypertension management in the community.

Nurses were pivotal as a source of hypertension health (57%) while only 12% reported that they would approach VHWs. The inclusion of VHW in the hypertension community care with be important in strengthening primary prevention of hypertension. A high defaulter rate of 31% was possibly due to recurrent stock-outs of antihypertensive medicines at the local clinic and lack of community tracking by the VHWs. Shortages of medication coupled with long travelling distances to the health facility contributed to poor hypertension outcomes; these findings are reported in other studies as well [28–30]. These challenges needed be addressed through primary prevention health education strategies on; treatment compliance, side effects and hypertension complications while making use of VHWs in community hypertension care.

In Zimbabwe there are limited national studies on hypertension prevalence while there is lack of infrastructure to enable and support hypertension surveillance [12]. This was evident in that more than 30% of respondents had last checked their blood pressure for more than 4 months while some had lost track of when they had a BP checked. The local clinic was the only place where a blood pressure machine was found however, sometimes the services would be unavailable to various logistical reasons. This then calls for concerted efforts to prioritize service delivery, and funding for hypertension consumables. Special priority and focus should be on the crafting policy and research-based implementation of tailor-made service delivery packages to reduce hypertension related morbidity and mortality [28].

The study had several limitations as there is no standardized instrument to measure hypertension knowledge, attitudes and practices. We therefore used literature, community knowledge and field experiences to design our data collection tools which may not have been exhaustive. The algorithm used for data collection left some chance of missing hypertensive patients or enrolling patients who may not have been diagnosed of hypertension. It is possible that recruitment bias could have been introduced in that all participants were self-reported hypertensive patients and there was no rigorous verification of hypertension diagnosis.

The study findings were used to identify gaps in knowledge, attitudes and practices including myths and misconceptions by hypertensive patients and the community at large on hypertension. These were then used to develop the methodology for the implementation phase of the community participatory action research (CBPR) study we conducted (these study findings were published in a separate paper) [31]. The CIG validated the study findings, and they then used these findings during the action reflection cycles for planning and learning purposes. Subsequently, the implementation of new community strategies for improved primary prevention of hypertension in the CBPR study [31], were informed by the findings in this baseline quantitative study. Thus, by implication recommendations of improved service delivery from the hypertension CBPR study were influenced partly by the findings in this study.

## Conclusion

Members of the community had poor knowledge on hypertension. This was associated with a lack of education and with strong beliefs in herbal and traditional medicines in the community, which influenced attitudes and practices on hypertension. Dietary risk factors were linked to poor knowledge. Hypertensive medicine shortages at the clinic resulted in worsened hypertension care and poor hypertension outcomes in the community.

## Supporting information

S1 File English questionnaire. (DOCX)

S2 File Translated Ndebele questionnaire. (DOCX)
