# Supplementary material for: Knowledge, attitudes and practices related to hypertension among residents of a disadvantaged rural community in southern Zimbabwe
Source: PLoS One. 2019 Jun 25;14(6):e0215500. doi: 10.1371/journal.pone.0215500 (PMC6657811; doi:10.1371/journal.pone.0215500)
Supplement: S1 File — (DOCX) [file pone.0215500.s001.docx]

#
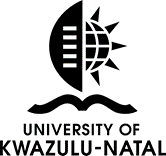


# University of KwaZulu Natal, Discipline of Public Health Medicine

# School of Nursing and Public Health

**Title:** Hypertension awareness, treatment and control in Gwanda district, Matebeleland South province, Zimbabwe; a participatory action research project.

# English Questionnaire to be used for baseline Phase 1 study.

| **Introduction**  Good Morning/Afternoon. My name is__________________________________________________  Our Principal Investigator is Dr Pugie Chimberengwa, from the Ministry of Health and Child Care. You are being invited to consider participating in a study: **Hypertension awareness, treatment and control in Gwanda district, Matebeleland south province, Zimbabwe; a participatory action research.** This research will involve you answering questions that I will ask you as guided by the questionnaire that I have. I would greatly appreciate if you spare me about 20 minutes of your time to go through the questionnaire. I shall be asking you a number of questions pertaining to high blood pressure. All the data collected will be treated with strict confidentiality and anonymity. If you feel that you cannot continue participating in the study, you are free to withdraw at any stage of the interview. Thanking you for your cooperation.  **Questionnaire number**: ……………. **Date**: …………………………………. | | | |
| --- | --- | --- | --- |
| **Socio-demographic Data** | | | |
| **No** | **Question** | **Options** | **Code** |
| **1** | How old are you | Number of completed years ……. |  |
| **2** | Gender (observe) | Male | 1 |
|  |  | Female | 2 |
| **3** | Marital status | Single | 1 |
|  |  | Married | 2 |
|  |  | Divorced | 3 |
|  |  | Widowed | 4 |
|  |  | Other (specify)….. | 77 |
| **4** | What is your religion | Christianity | 1 |
|  |  | African tradition | 2 |
|  |  | Apostolic (specify) …. | 4 |
|  |  | Other (specify) …. | 77 |
| **5** | Does your religion permit you to seek modern medical assistance? | Yes | 1 |
|  |  | No | 2 |
| **6** | What is your highest educational level? | None | 1 |
|  |  | Primary | 2 |
|  |  | Secondary | 3 |
|  |  | Tertiary | 4 |
| **7** | What is your occupation? | Job…………………. |  |
| **8** | Job description | Skilled | 1 |
|  |  | Unskilled | 2 |
| **9** | Family monthly income in US$ | <100 | 1 |
|  |  | 100-300 | 2 |
|  |  | >300 | 3 |
| **Lifestyle Related Factors (physical activity, diet, alcohol consumption and smoking)** | | | |
| **10** | Have you ever consumed alcohol*?* | Yes | 1 |
|  |  | No *(skip to Qn.12)* | 2 |
| **11** | In the past 12 months, have you frequently taken alcohol? | 5 or more days/week | 1 |
|  |  | 1-4 days/week | 2 |
|  |  | 1-3 days/month | 3 |
|  |  | Less than once a month | 4 |
| **12** | Have you ever smoked (cigarette, snuff, pipe, chew) tobacco*?* | Yes | 1 |
|  |  | No *(skip to Qn.16)* | 2 |
| **13** | Do you currently smoke? | Yes | 1 |
|  |  | No *(skip to Qn.15*) | 2 |
| **14** | On average, how many of the following do you smoke per day*.* | Manufactured cigarettes………... | 1 |
|  |  | Hand rolled cigarettes ……… | 2 |
|  |  | Tobacco pipe filling …………. | 3 |
| **15** | If currently non-smoker, did you regularly smoke in the past? | Yes | 1 |
|  |  | No | 2 |
| **16** | In a typical week, how many days do you eat fruits? | No. of days  ………………… |  |
| **17** | In a typical week, how many days do you eat vegetables? | No of days…… |  |
| **18** | Do you often add salt to your food on the table? | Yes | 1 |
|  |  | No | 2 |
| **19** | What type of oil/fat is mostly used to prepare food at your home? | Vegetable oil | 1 |
|  |  | Animal fat | 2 |
|  |  | Margarine | 3 |
|  |  | Peanut butter | 4 |
|  |  | None | 5 |
| **20** | What form of exercise or manual work do you do? (elaborate) | |  |
|  |  | |  |
|  |  | |  |
|  |  | |  |
|  |  | |  |
| **Family Related Factors** | | | |
| **21** | Is there anyone in your family (parents or siblings) who suffered / has hypertension? (elaborate) | |  |
|  |  | |  |
|  |  | |  |
|  |  | |  |
|  |  | |  |
| **22** | Is there anyone in your family who has/had the following complications of hypertension? (heart failure, stroke, kidney failure) | |  |
|  |  | |  |
|  |  | |  |
|  |  | |  |
|  |  | |  |
|  |  |  |  |
|  |  |  |  |
| **Knowledge on Hypertension Awareness, Treatment and Control** | | | |
|  |  |  | |
| **23** | What is your comment on the use of antihypertensive medicines/tablets on lowering blood pressure | | |
|  |  | | |
|  |  | | |
|  |  | | |
|  |  | | |
| **24** | What is your comment on the use of traditional remedies used to treat hypertension (high blood pressure)? | |  |
|  |  |  |  |
|  |  | |  |
|  |  | |  |
|  |  | |  |
|  |  | |  |
| **25** | Would you use traditional medicines to control your blood pressure? | Yes | 1 |
|  |  | No | 2 |
| **26** | If you need more knowledge on high blood pressure (hypertension) where would you get it? | Local clinic nurse | 1 |
|  |  | Village health worker | 2 |
|  |  | Public hospital | 3 |
|  |  | Other(specify)  ……………………......... | 77 |
| **27** | Has any health worker / village health worker discussed with you about blood pressure treatment and control? How would you rate your knowledge? elaborate | |  |
|  |  |  |  |
|  |  |  |  |
|  |  |  |  |
|  |  |  |  |
|  |  |  |  |
|  |  |  |  |
| **28** | How would you define high blood pressure? | |  |
|  |  |  |  |
|  |  |  |  |
|  |  |  |  |
|  |  |  |  |
| **29** | How would you know that your blood pressure is high? | |  |
|  |  | |  |
|  |  | |  |
|  |  | |  |
| **30** | What causes high blood pressure (hypertension)? | Unknown | 1 |
|  |  | Drugs | 2 |
|  |  | Witchcraft | 3 |
|  |  | Old age | 4 |
|  |  | Stress | 5 |
|  |  | Other (specify)……. | 77 |
| **31** | What are the signs and symptoms of high blood pressure? | asymptomatic | 1 |
|  |  | headache | 2 |
|  |  | palpitations | 3 |
|  |  | Poor vision | 4 |
|  |  | Dizziness | 5 |
|  |  | Other (specify)……… | 77 |
| **32** | Can one have high blood pressure (hypertension) without any signs and symptoms? | Yes | 1 |
|  |  | No | 2 |
| **33** | What can happen if blood pressure remains untreated*?* | stroke | 1 |
|  |  | Heart failure | 2 |
|  |  | Kidney failure | 3 |
|  |  | Loss of sight | 4 |
|  |  | Death | 5 |
|  |  | Don’t know | 99 |
| **34** | What are the risk factors of developing high blood pressure? | Hereditary | 1 |
|  |  | Smoking | 2 |
|  |  | Obesity | 3 |
|  |  | High fat intake | 4 |
|  |  | Excess alcohol intake | 5 |
|  |  | High salt intake | 6 |
|  |  | Don’t know | 99 |
| **35** | How can you prevent/ control hypertension?  ……………………………………………………..  ………………………………………………………  ……………………………………………………….  ………………………………………………………..  ………………………………………………………. | Minimize salt intake | 1 |
|  |  | Reduce fatty foods | 2 |
|  |  | Avoid excess alcohol | 3 |
|  |  | Avoid smoking | 4 |
|  |  | Regular exercise | 5 |
|  |  | Taking antihypertensive | 6 |
|  |  | Other (state)  ………………………… | 77 |
|  |  | Don’t know | 99 |
| **36** | In your own opinion, why do people not like taking/ default blood pressure treatment? | |  |
|  |  | |  |
|  |  | |  |
|  |  | |  |
|  |  | |  |
| **Treatment and Control of Hypertension** | | | |
| **37** | How do you currently control your blood pressure? | Blood pressure tablets | 1 |
|  |  | Traditional medicines | 2 |
|  |  | Other (state)  ……………………………. | 77 |
| **38** | Are you currently taking medication for hypertension? | Yes | 1 |
|  |  | No *(skip to Qn. 47)* | 2 |
| **39** | If yes, have you taken medication regularly in the past 2 weeks? | Always regularly taking | 1 |
|  |  | Intermittently as needed | 2 |
| **40** | Do you have any challenges with taking hypertensive treatment? explain | |  |
|  |  | |  |
|  |  | |  |
|  |  | |  |
|  |  | |  |
| **41** | Where do you prefer being followed up for hypertension management? Why?  ……………………………………………………..  ………………………………………………………  ……………………………………………………….  ………………………………………………………..  ………………………………………………………. | Local clinic | 1 |
|  |  | Private doctor | 2 |
|  |  | Public hospital | 3 |
|  |  | Other (specify)…… | 77 |
| **42** | When last was your blood pressure checked? | < 1 month ago | 1 |
|  |  | - 1. months ago | 2 |
|  |  | >4 months ago | 3 |
| **43** | Is your blood pressure well controlled? | Yes | 1 |
|  |  | No | 2 |
|  |  | Don’t know | 99 |
| **44** | What medication are you taking for hypertension*?* State | |  |
|  |  | |  |
|  |  | | |
|  |  | | |
|  |  | |  |
| **45** | Have you ever defaulted treatment? | Yes | 1 |
|  |  | No (*skip to Qn. 55)* | 2 |
| **46** | Why did you default?  ……………………………………………………..  ………………………………………………………  ……………………………………………………….  ………………………………………………………..  ………………………………………………………. | Feeling much better | 1 |
|  |  | Tablets make me sick | 2 |
|  |  | Treatment is not effective | 3 |
|  |  | To avoid addiction | 4 |
|  |  | Developed side effects | 5 |
|  |  | Was trying alternative remedies | 6 |
|  |  | Other (specify)……. | 77 |
| **Perceptions and Recommendations** | | | |
| **47** | Would you visit a traditional healer/ prophet for hypertension related illnesses? | |  |
|  |  | |  |
|  |  | |  |
|  |  | |  |
| **48** | What challenges do you face as a person living with hypertension in the community | |  |
|  |  | |  |
|  |  | |  |
|  |  | |  |
|  |  | |  |
| **49** | In your personal view, how can these challenges be addressed by the health department and the community? | |  |
|  |  | |  |
|  |  | |  |
|  |  | |  |
|  |  | |  |
|  |  | |  |
|  |  | |  |
| **50** | What other issues would you want to highlight or discuss pertaining to hypertension? | |  |
|  |  | |  |
|  |  | |  |
|  |  | |  |
|  |  | |  |
|  |  | |  |
