# Supplementary material for: Knowledge, attitudes and practices related to hypertension among residents of a disadvantaged rural community in southern Zimbabwe
Source: PLoS One. 2019 Jun 25;14(6):e0215500. doi: 10.1371/journal.pone.0215500 (PMC6657811; doi:10.1371/journal.pone.0215500)
Supplement: S2 File — (DOCX) [file pone.0215500.s002.docx]

**
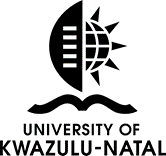
**

**University of KwaZulu Natal****, Discipline of Public Health Medicine**

**School of Nursing and Public Health**

**Title:** Hypertension awareness, treatment and control in Gwanda district, Matebeleland South province, Zimbabwe; a participatory action research project.

**Ndebele Questionnaire to be used for baseline Phase 1 study.**

| **Isingenso**  Ikuseni/Imini Enhle. Igama lami ngingu ________________________________  Umphathi wethu ophezulu wokucubungula ngu Dokotela Pugie Chimberengwa, wo gatsha lwezempilakahle lokukhangela ukuphathwa kwabantwana. Uyanxuzwa ukuphatheka kuloluhlelo lokucubungula: Unkhankaso we BP**, Ukwelatshwa lokunanzelela eGwanda district, ese Matebeleland south province, eZimbabwe; ukuphatheka ekuhlubunguleni** Lokhukucubungula kuzahlanganisa wena uphendula limbuzo esizakubuza yona siqondiswa yimibuzo le esilayo. Singathokoza uma ungasinika imizizwana engamatshumi amabiliyesikhathi sakho sikhangele limbuzo. Ngizabe ngikubuza imibuzo thile mayelana le BP. Yonke impendulo izaphathwa ngoku fihlakala lokungaziwa ukuthi itholakale ivela kubani. Uma ubona angani ungeke wenelise ukuqhubekela phambili ngokuphatheka kulimbuzo ukhululekile ukuyekela kuloba kusiphi isigaba sokubuza.. Siyabonga ngokuphatheka kwakho.  **Imbuzo Nombolo** : ……………. **Ilanga**: …………………………………. | | | |
| --- | --- | --- | --- |
| **Socio-demographic Data** | | | |
| **No** | **Umbuzo** | **Khetha** | **Code** |
| **1** | Uleminyaka emingaki | Iminyaka osuyigoqile ……. |  |
| **2** | Umhlobo (Khangela) | Owesilisa | 1 |
|  |  | Owesifazane | 2 |
| **3** | Isimo somtshado | Uzimele wedwa | 1 |
|  |  | Utshadile | 2 |
|  |  | Usuke emtshadweni | 3 |
|  |  | Wafelwa | 4 |
|  |  | Olunye (chasisa)….. | 77 |
| **4** | Ukholwani | Isikristu | 1 |
|  |  | Isintu | 2 |
|  |  | Isipostoli (chasisa) …. | 4 |
|  |  | Eyinye (chasisa) …. | 77 |
| **5** | Ukholo lwakho luyakuvumela ukudinga usizo kwabezempilakahle ? | Yebo | 1 |
|  |  | Hatshi | 2 |
| **6** | Wacina kuliphi ibanga lezemfundo? | Akula | 1 |
|  |  | Ungwalo lwangaphansi | 2 |
|  |  | Ugwalo lwemfundo yaphezulu | 3 |
|  |  | Ugwalo lwemfundo ephezulu | 4 |
| **7** | Usebenza ni? | Umsebezi…………………. |  |
| **8** | Incazelo yomsebezi | Ucebile | 1 |
|  |  | Awucebanga | 2 |
| **9** | Inzuzo yenyanga ngama US$ | <100 | 1 |
|  |  | 100-300 | 2 |
|  |  | >300 | 3 |
| **Okuphathelene lokuphila kwakho ( Okudliwayo, Okudakayo lokubhemnywayo )** | | | |
| **10** | Sewake wanatha okudakayo*?* | Yebo | 1 |
|  |  | Hatshi *(Yeqa uye kumbuzo no.12 )* | 2 |
| **11** | Kunyanga ezilitshumi lambili ezidluleyo uke wanatha okudakayo? | Alamalanga amahlanu kumbe adlulayo/kumbe iviki | 1 |
|  |  | Ilanga elilodwa kusiya kwamane ngeviki | 2 |
|  |  | Ilanga elilodwa kusiya amathathu nge nyanga | 3 |
|  |  | Okungaphansi kokukodwa ngenyanya | 4 |
| **12** | Sowake wabhema okudakayo (Igwayi, elamakhala, elephayiphi, kumbe elihlafunwayo) | Yebo | 1 |
|  |  | Hatshi *(Yeqa uye kumbuzo no 16)* | 2 |
| **13** | Uyake ubheme kwezinye izikhathi? | Yebo | 1 |
|  |  | Hatshi *(yeqa uye ku no. 15*) | 2 |
| **14** | Ungabe uthatha okunganani okudakayo ngelanga. | Okubhenywayo okwenziweyo ………... | 1 |
|  |  | Okubhenywayo Okugoqwayo ……… | 2 |
|  |  | Okubhenywayo ngephiyiphi …………. | 3 |
| **15** | Ma ungabhemi khathesi kodwa wake wabhema empilweni yakho? | Yebo | 1 |
|  |  | Hatshi | 2 |
| **16** | Ngeviki udla kangaki izithelo (Fruits)? | Inani lamalanga ………………… |  |
| **17** | Evikini udla kangaki imibhida? | Inani lamalanga  …… |  |
| **18** | Uyafaka umunyu ekudleni kwakho eganwini? | Yebo | 1 |
|  |  | Hatshi | 2 |
| **19** | Yiwuphi umhlobo wamafutha asetshenziswa ekuphekeni ngekhaya? | Amafutha emibhida | 1 |
|  |  | Amafutha enyamazana | 2 |
|  |  | uMargarine | 3 |
|  |  | Idobi | 4 |
|  |  | Akula | 5 |
| **20** | Ungachaza imisebenzi yakho yansuku zonke kumbe lokuhamba hamba | |  |
|  |  | |  |
|  |  | |  |
|  |  | |  |
| **Okuphathelane lemuli** | | | |
| **21** | Engaba khona emulini (Umzali kumbe abantwana ongabe egula umkhuhlane kumbe olomkhuhlane wo BP? chasisa | |  |
|  |  | |  |
|  |  | |  |
|  |  | |  |
| **22** | Engaba khona emulini ongaba lale imikhuhlane elandelayo ehambelanayo le BP? Umkhuhlane wenhliziyo, iStroke , umkhuhlane wezinso, lomkhuhlane we tshukela? | |  |
|  |  | |  |
|  |  | |  |
|  |  | |  |
|  |  | |  |
| **Ulwazi nge BP, ukukhankasa, ukwelatshwa lokunanzelela.** | | | |
| **23** | Uyakholwa ukuthi amaphilisi e BP ayasebenza ukwehlisa ukugijima kwegazi . (chasia imibono yakho) | |  |
|  |  | |  |
|  |  | |  |
|  |  | |  |
| **24** | Ungabe usazi okwesintwini okusetshenziswa ukwelapha iBP? Uthini ngakho? | |  |
|  |  | |  |
|  |  | |  |
|  |  | |  |
| **25** | Ungasebenzisa okwesintwini ukukhangela I BP yakho? | Yebo | 1 |
|  |  | Hatshi | 2 |
| **26** | Ma ufuna ulwazi nge BP ungaluthola ngaphi? | Umongikazi oseclinic eduzane | 1 |
|  |  | Kwabezempilakahle esigabeni | 2 |
|  |  | Esibhedlela sikazulu | 3 |
|  |  | Kwezinye indawo (Chasisa)  ……………………......... | 77 |
| **27** | Kungaba labezempilakahle kumbe abasesigabeni bemele ezempilakahle bake bakhulumisana lawe ngomkhuhlane we BP ukwelatshwa kwayo loku nanzelela? Chaza ulwazi lwakho | |  |
|  |  | |  |
|  |  | |  |
|  |  | |  |
|  |  | |  |
| **28** | Ungachaza kanjani ukuthi i BP ngumkhuhlane bani? | |  |
|  |  | |  |
|  |  | |  |
|  |  | |  |
| **29** | Ungazi kanjani ukuthi IBP yakho ikhwelile? | |  |
|  |  | |  |
|  |  | |  |
|  |  | |  |
| **30** | Kuyini okubangela I BP? | Unknown | 1 |
|  |  | Drugs | 2 |
|  |  | Witchcraft | 3 |
|  |  | Old age | 4 |
|  |  | Stress | 5 |
|  |  | Other (specify)……. | 77 |
| **31** | Yiziphi izitshengiselo ze BP? | asymptomatic | 1 |
|  |  | Headache | 2 |
|  |  | palpitations | 3 |
|  |  | Ukungaboni kuhle | 4 |
|  |  | Isiyezi | 5 |
|  |  | Okunye (Chasisa)……… | 77 |
| **32** | Umuntu engaba le BP engela zibonakaliso? | Yebo | 1 |
|  |  | Hatshi | 2 |
| **33** | Kungenzakalani uma I BP ingelatshwanga? | Stroke | 1 |
|  |  | Umkhuhlane wenhliziyo | 2 |
|  |  | Umkhuhlane wezinso | 3 |
|  |  | Ukulahlekelwa yikubona | 4 |
|  |  | Ukufa | 5 |
|  |  | Angazi | 99 |
| **34** | Ngokwazi kwakho, kuyini okungenza ukuthi umuntu abe sezingeni eliphezulu lokuba lomkhuhlane weBP | Kusegazini | 1 |
|  |  | Ukubhema | 2 |
|  |  | Ukunona | 3 |
|  |  | Ukudhla amafutha amanengi | 4 |
|  |  | Ukunatha okudakayo ngokungaphezulu | 5 |
|  |  | Ukuthatha usawudo ngokungaphezulu | 6 |
|  |  | Angazi | 99 |
| **35** | Singayenqabela kanjani I BP?  ……………………………………………………..  ………………………………………………………  ……………………………………………………….  ………………………………………………………..  ………………………………………………………. | Yehlisa ukuthatha istwayi | 1 |
|  |  | Yehlisa ukudla okulamafutha | 2 |
|  |  | Yenqaba okudakayo ngokudlulisileyo | 3 |
|  |  | Yenqaba ukubhema | 4 |
|  |  | Ukuzelula njalonjalo | 5 |
|  |  | Taking antihypertensive | 6 |
|  |  | Okunye (Kuqambe)  ………………………… | 77 |
|  |  | Angazi | 99 |
| **36** | Ngokubona kwakho, kungani abantu bengathandi/ ukuthatha amaphilisisi e BP? | |  |
|  |  | |  |
|  |  | |  |
| **Ukwelapha lokunanzelela iBP** | | | |
| **37** | Unakekela kanjani I BP yakho? | Amaphilisi e BP | 1 |
|  |  | Imithi yesintwini | 2 |
|  |  | Okunye (Tshono) ……………………………. | 77 |
| **38** | Uyathatha amaphilisi e BP? | Yebo | 1 |
|  |  | Hatshi *(Yeqa uye kumbuzo 47)* | 2 |
| **39** | Ma unatha ubuthatha amaphilisi kanjani okwamaviki amabili edluleyo? | Bengithatha njalonjalo | 1 |
|  |  | Bengithatha ma esefunakala | 2 |
| **40** | Ulohlupho na lokunatha amapilisi awe BP? Chasisa | |  |
|  |  | |  |
|  |  | |  |
|  |  | |  |
| **41** | Ufisa ukulandelelwa ngaphi ekunanzweni iBP? Izizatho zakho ngezani?  ……………………………………………………..  ……………………………………………………… | Ekilinika esiseduzane | 1 |
|  |  | KuDokotela wami | 2 |
|  |  | Esibhedlela sikazulu | 3 |
|  |  | Kwezinye (chasisa)…… | 77 |
| **42** | Wacina nini ukukhangelwa iBP? | Ngaphansi kwenyanga eyodwa | 1 |
|  |  | Enyangeni ezimbili kusiya kwezine | 2 |
|  |  | Kwezine kusiyaphezulu | 3 |
| **43** | IBP yakho iyananzelelwa yini? | Yebo | 1 |
|  |  | Hatshi | 2 |
|  |  | Angazi | 99 |
| **44** | Uthatha maphilisi bani awe BP? Atsho  ……………………………………………………..  ………………………………………………………  ………………………………………………………. | |  |
| **45** | Sewake waqisa ukuthatha amaphilisi? | Yebo | 1 |
|  |  | Hatshi (Yeqa uyeku mbuzo wesi  *55)* | 2 |
| **46** | Kungani wama?  ……………………………………………………..  ………………………………………………………  ……………………………………………………….  ………………………………………………………..  ………………………………………………………. | Ngasengingcono | 1 |
|  |  | Imbumbuluza zenza ngigule | 2 |
|  |  | Amaphilisi awaphathisi | 3 |
|  |  | Ukwenqabela ukungingena egazini | 4 |
|  |  | Ukuqala okungahambelani lami | 5 |
|  |  | Bengizama ezinye indlela | 6 |
|  |  | Okunye (chasisa) ……. | 77 |
| **Okukhangelelweyo lesizakwenza** | | | |
| **47** | Ungayana enyangeni kumbe kubaphorofitha ngokuphathelene le BP? chasisa | |  |
|  |  | |  |
|  |  | |  |
|  |  | |  |
| **48** | Yiziphi inhlupho ohlangana lazo njengomuntu olomkhuhlane we BP esigabeni | |  |
|  |  | |  |
|  |  | |  |
|  |  | |  |
| **49** | Ngombono wakho, lezi nhlupho zingalungisiswa kanjani emphakathini kuncediswana labezempilakahle? | |  |
|  |  | |  |
|  |  | |  |
|  |  | |  |
| **50** | Kungaba lokunye na esingaxoxa ngakho engingabe ngingakubuzanga okuphatelane le BP? | |  |
|  |  | |  |
|  |  | |  |
|  |  | |  |
|  |  | |  |
